# Hydrogen in *α*-iron: role of phonons in the diffusion of interstitials at high temperature

**DOI:** 10.1038/s41598-019-48490-w

**Published:** 2019-08-20

**Authors:** Pedro L. de Andres, Javier Sanchez, Alvaro Ridruejo

**Affiliations:** 1 Instituto de Ciencia de Materiales de Madrid-CSIC, Madrid, E-28049 Spain; 2 Instituto de Ciencias de la Construcción Eduardo Torroja-CSIC, Madrid, E-28033 Spain; 3 Departamento de Ciencia de Materiales, ETSI Caminos Canales y Puertos, Universidad Politécnica de Madrid, Madrid, E-28040 Spain

**Keywords:** Thermodynamics, Electronic properties and materials, Civil engineering

## Abstract

Ab-initio Density Functional Theory has been used to compute phonons for interstitial hydrogen in *α*-iron. In the harmonic approximation, these phonons yield Helmholtz’s free energy as a function of temperature, which can be used to obtain diffusion barriers from an Arrhenius plot. By comparing with the experimental database compiled by Kiuchi and McLellan, we show that the role of phonons is crucial to understand the diffusion of interstitial hydrogen at T > 300 K. The computed specific heat for Fe_16_H and Fe behaves quite differently due to the appearance of optical modes and could be used to calibrate the amount of interstitials in the iron matrix.

## Introduction

Civil engineering structures like buildings, bridges, dams, and others, rely on cores of ferritic steels made of body-centred-cubic (bcc) iron (*α*-Fe). To understand structural performances, it is of paramount importance to study the conditions that may degrade their properties, like mechanical strength, ductility, toughness, etc. Hydrogen embrittlement is arguably the most frequent cause for catastrophic failure in structures subjected to tensile loads and consequently, one of the most active research areas in metallurgy^1–8^. High strength ferritic steels are particularly vulnerable to hydrogen embrittlement, which despite having been first reported as early as in 1875^9^, is a complex phenomenon that has so far evaded a fully satisfactory explanation. There are currently several promising strategies trying to describe, understand and link the intervening mechanisms at several scales (multiscale approach)^3,10^. Likewise, many experimental studies have adopted the same approach and characterised these mechanisms from the atomic scale to the macroscopic one^6,11–13^.

Among the relevant phenomena, hydrogen transport in the bcc iron lattice plays a prominent role in hydrogen embrittlement. This process has been addressed both from the theoretical and experimental viewpoints. First-principles calculations show the stability of the cubic lattice high symmetry points (tetrahedral and octahedral, respectively) to host interstitial hydrogen and the associated diffusion barriers^14–18^. There are many experimental results to determine the diffusion coefficient of hydrogen in *α*-Fe^19,20^. However, lattice defects trap hydrogen and affect these measurements, which can only be interpreted as an effective diffusion coefficient. Analysis of thermal desorption of hydrogen is the method of choice to characterise the influence of lattice defects^21–23^. Since this technique also depends on the value of the diffusion coefficient of H in the bcc iron lattice, a realistic measurement of the diffusion coefficient can be obtained through successive iterations. In this regard, a reasonable initial theoretical estimate of the defect-free diffusion coefficient over an extended temperature range is of great importance to decouple observed diffusion from the effect of H traps. Such an estimation will prove valuable for the interpretation of desorption experiments and to feed computational models for diffusion (such as standard or Kinetic Monte Carlo models).

The goal of this paper is to study from first-principles the contribution of phonons to the diffusion of hydrogen in *α*-Fe. Recent work by different groups has shown that ab-initio spin-polarised density functional theory (DFT)^24,25^ based on pseudopotentials^26–28^ is a reliable tool to describe the physical properties of iron, including structural parameters, magnetic properties, elastic constants, and different phases^14–16,18,29^. Phonons for *α*-Fe with interstitial H yield the vibrational internal free energy, allowing to extend previous DFT studies to the *T* ≠ 0 K domain and to probe the influence of temperature on the diffusion of hydrogen. As shown, phonon contributions are a crucial factor to explain the diffusion of interstitial hydrogen at high temperature.

## Results

To study the effect of phonons on the diffusion of interstitial hydrogen we set up a model based on a 2 × 2 × 2 body-centred cubic unit cell (Fe16), which is large enough to minimise the interaction of the interstitial with its periodic-generated images, and at the same time, it is amenable for the task of computing phonons. According to the precision of our DFT calculations (c.f. Methods: Density Functional Theory) the optimum cubic cell has *a* = *b* = *c* = 5.668 Å, *α* = *β* = *γ* = 90°, symmetry group IM-3M (IT # 229), and a magnetic moment per atom of 2.18 *μ*_*B*_. Accepted experimental values for these magnitudes are 5.734 Å and 2.22 *μ*_*B*_^30^; therefore, the fractional errors in these calculations are 1.2% and 1.8% respectively, which show the adequacy of the theoretical setup for our purposes.

### Tetrahedral and octahedral high-symmetry sites

Optimization of forces and stresses for a hydrogen concentration Fe_16_H results in a global minimum where the interstitial is located at the tetrahedral high-symmetry site. We recall that for large concentrations of interstitials, the internal stresses originated by the lattice distortions needed to accommodate these interstitials alter the relative equilibrium between tetrahedral and octahedral sites, making the octahedral site the global minimum^16^. Therefore, it is clear that for the task of finding a global energy minimum, both forces and stresses have to be minimized in a consistent way. We find that the internal stress related to the tetrahedral interstitial is accommodated in the cubic unit cell by the following internal deformation: *a* = *b* = 5.751 Å, *c* = 5.747 Å, *α* = *β* = *γ* = 90°, symmetry group P-4M2 (IT # 115), a magnetic moment per atom of 2.21 *μ*_*B*_, and residual stresses *σ*_*xx*_ = *σ*_*yy*_ = −0.002, *σ*_*zz*_ = 0.02 GPa. For the octahedral site, a similar optimization of forces and stresses results in an stationary point (unstable) with *a* = *b* = 5.60 Å, *c* = 5.894 Å, *α* = *β* = *γ* = 90° symmetry group P4/MMM (IT # 123), a magnetic moment per atom of 1.93 *μ*_*B*_, and *σ*_*xx*_ = *σ*_*yy*_ = 0.001, *σ*_*zz*_ = 0.002 GPa. A Hirshfeld analysis for the stable tetrahedral site reveals that H attracts a small amount of negative charge (−0.07 e) but carries a negligible amount of spin (0.01 *μ*_B_).

### Phonons

**Figure 1:**
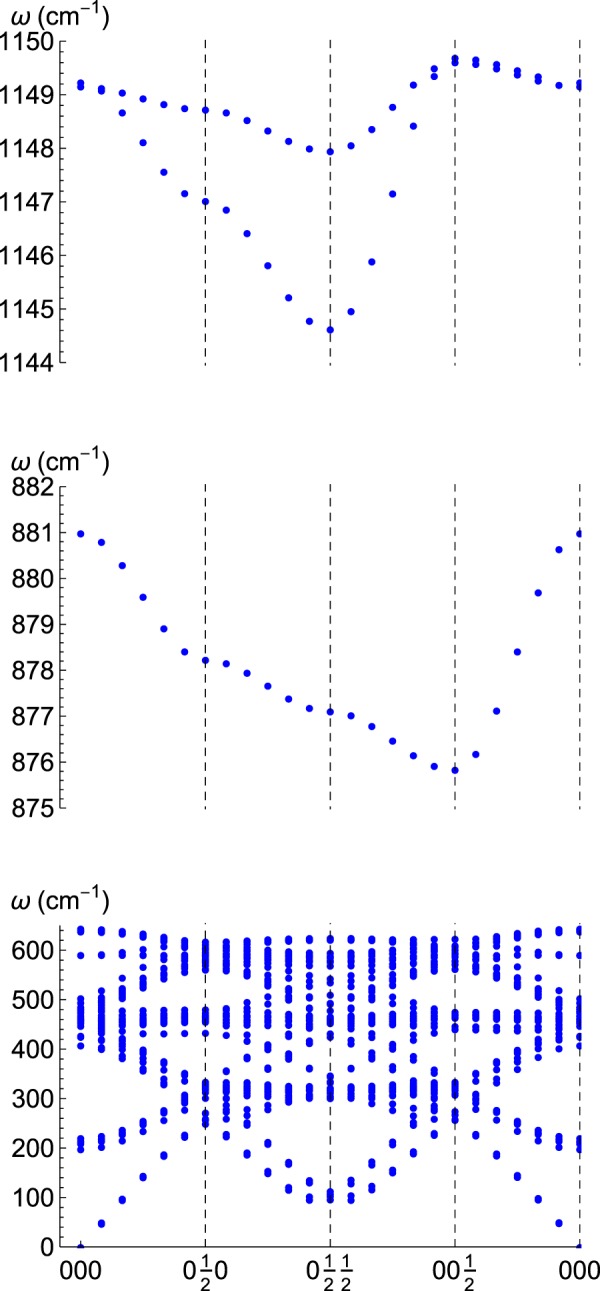
Phonon spectra along a closed path in the Brillouin zone for interstitial H in a tetrahedral site in the 2 × 2 × 2 bcc unit cell.

**Figure 2:**
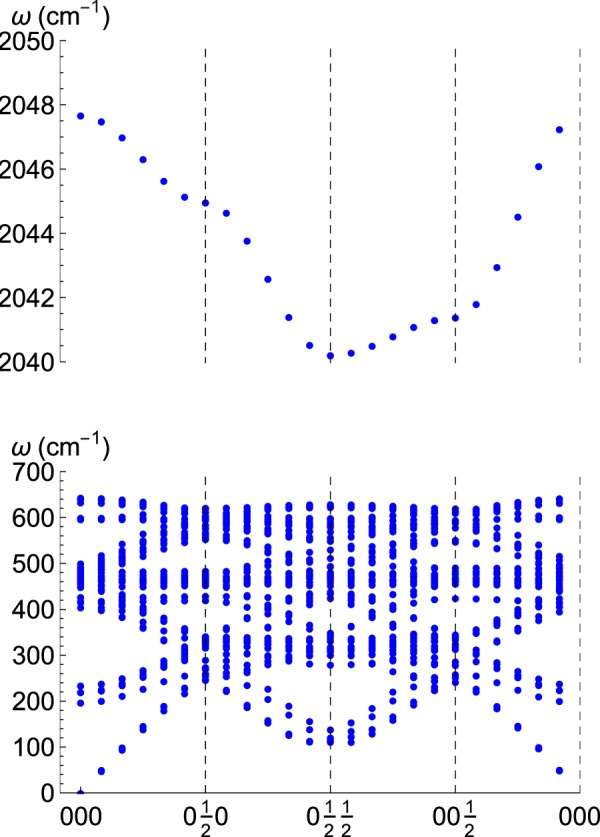
Phonon spectra along a closed path in the Brillouin zone for interstitial H in a octahedral site in the 2 × 2 × 2 bcc unit cell.

**Figure 3:**
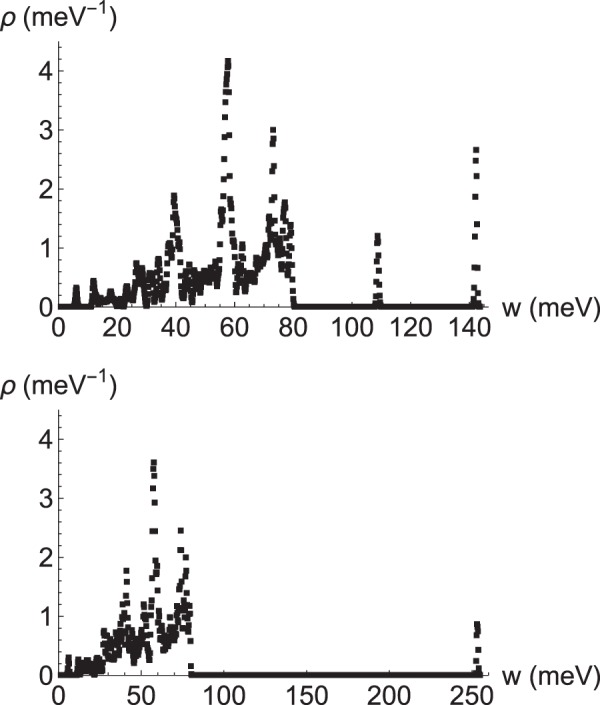
Phonon density of states for interstitial H in a tetrahedral site (upper panel, ${\int }_{0}^{\infty }\,\rho (w)dw=50.8$) and in the octahedral site (lower panel, ${\int }_{0}^{\infty }\,\rho (w)dw=48.7$).

The main result of our calculations are phonon spectra for Fe_16_H with (i) the interstitial located in a tetrahedral site (Fig. 1), or (ii) in an octahedral site (Fig. 2). Figure 3 gives the corresponding density of states, *ρ*(*w*), which allows us to obtain the thermodynamical magnitudes listed in the section of Methods (Thermophysics). When comparing with pristine bcc-Fe, the significant feature of these spectra is the appearance of high-frequency optical modes. Regarding the internal electronic energy, the tetrahedral site corresponds to a stable stationary point; therefore, three vibrational modes appear approximately between 900 and 1200 cm^−1^. On the other hand, the octahedral site represents an unstable point (local maximum) with two imaginary modes, and one real with *w* ≈ 2045 cm^−1^. These are related to Fe-H vibrations, as a gaussian calculation confirms for FeH: this simple model has a single stretching frequency at 1780 cm^−1^ for an equilibrium distance of 1.57 Å between H and Fe (to be compared with 1.47 Å, the distance between H in the octahedral site and the nearest Fe)^31^.

**Figure 4:**
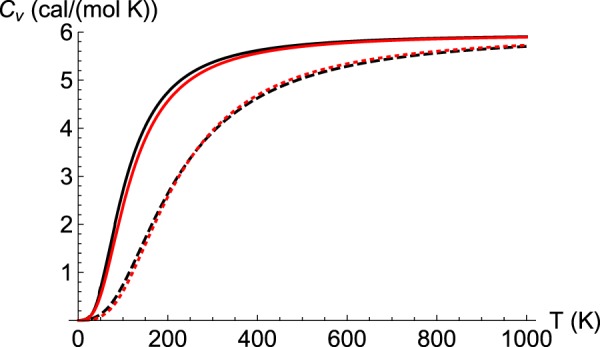
Constant volume specific heat for *α*-Fe (black continuous) and Fe_16_H (black discontinuous). These ab-initio calculations are well represented by Debye’s model with *T*_*D*_ = 476 K (red continuous, *α*-Fe), and *T*_*D*_ = 900 K (red discontinuous, Fe_16_H).

Physical insight on the role of these new optical modes as a function of T can be obtained by computing the specific heat at constant volume (*C*_*V*_(*T*), Equation (9). By comparing with pristine bcc-Fe, we see how the main effect of high-frequency optical modes is to shift *C*_*V*_ towards higher values of temperature, e.g. compare black dashed (Fe_16_H) and black continuous (Fe) in Fig. 4. This result constitutes a precise prediction that can both be tested with experiments, and can also be used to calibrate the number of interstitials due to its sensitivity to concentration. While a Debye’s model represents well pristine Fe with an acceptable value *T*_*D*_ = 476 K^32^, we observe that Fe_16_H demands a higher value, *T*_*D*_ = 900 K, which usually is interpreted as a hardening of the material. However, we notice that in this case the higher value for *T*_*D*_ is more related to the appearance of optical modes than to the hardening of the acoustic ones; in practice, such a large value for *T*_*D*_ shows the difficulty to populate high-frequency modes at low temperatures. This interpretation is supported by a calculation of the elastic constants for Fe_16_H (for reference, ab-initio values for Fe computed with the same formalism are given in parantheses): *C*_11_ = 177(187), *C*_12_ = 87(76), *C*_44_ = 52(62) GPa. These values can be transformed into an averaged sound speed by using Debye’s theory, which could be useful to test the theory with experiments. We get 2.7 km/s for Fe_16_H, to be compared with the value 2.9 km/s for pure *α*-Fe (an experimental value for Fe is 3.6 km/s^32^). Therefore, we conclude that despite the need for a larger best-fit Debye temperature, a comparison with the elastic constants for the pristine material indicates that the presence of interstitials tends to make it softer.

For the process of diffusion, which is our primary concern, we turn now to the vibrational properties of Fe_16_H while H is near the transition state. The calculation of phonons properly converged right at the transition state was a challenging task due to the unstable nature of the transition state and its low symmetry. Therefore, we investigate the phonons at the nearby octahedral site, which is also unstable but belongs to a specific group symmetry that can be used to perform a constrained calculation. We have remarked on the importance of eliminating internal stresses to find equilibrium configurations. However, for the barriers in the diffusion process, we find that this is not a crucial point since the time spent by the interstitial near the barrier is not enough to allow a full relaxation of the whole lattice. Therefore, we compute energy configurations near the octahedral site using parameters for the periodic lattice which correspond to the global equilibrium configuration for the system, i.e., the tetrahedral site. In such a configuration the material is under the following internal stress: *σ*_*xx*_ = *σ*_*yy*_ = 1.3, *σ*_*zz*_ = −2.7 GPa. As expected, for such a non-stable configuration, we find that a doubly degenerated optical mode becomes soft, while a third optical branch hardens considerably, cf. compare Figs 1 and 2 (top panels). Such a high-frequency optical mode is responsible for a significant increased zero-point energy correction between the tetrahedral and the octahedral sites, which becomes a relevant feature for the diffusion of the interstitial, as we discuss in the next section.

**Figure 5:**
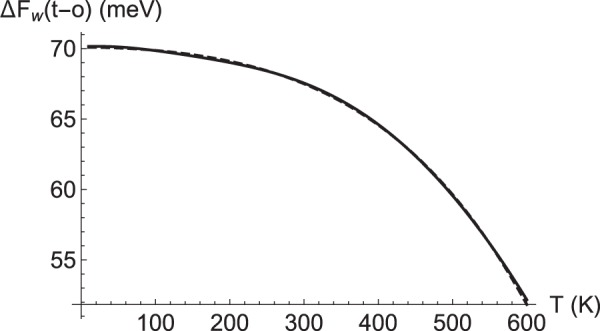
Helmholtz’s Free Energy difference between tetrahedral and octahedral sites (meV), as a function of *T* (K). The dashed curve is a polynomial approximation to the full calculation, Δ*F*_*w*_(*T*) = 70 − 3.9 × 10^−5^ *T*^2^ − 3.5 × 10^−11^*T*^4^, accurate enough to be used in the calculation of the diffusion coefficient.

Figure 5 shows the difference between Helmholtz’s free energies for tetrahedral and octahedral sites related only to phonons, obtained by using Equation (4), which gives us access to the variation with temperature of diffusion barriers (the electronic contribution to the free energy, which is temperature independent, is considered separately). The value Δ*F*(*t* − *o*, *T* = 0) gives the difference between the zero-point energy correction for both sites, +70 meV, which reduces the difference in the internal electronic energy between these two sites from −125 meV to −55 meV, a significant 55% correction. Such correction is almost entirely due to the different frequencies of optical phonons related to the occupation of tetrahedral (t) and octahedral (o) sites, as it can be shown by taking the difference between their respective values, *w*_*t*_ = 394 meV and *w*_*o*_ = 253 meV: $\frac{1}{2}({w}_{t}-{w}_{o})=71$ meV, which can be compared to the zero-point correction (*T* = 0 K) obtained from the difference in free energies: *F*_*t*_ − *F*_*o*_ = 70 meV. Therefore, both by the quantitative determination of the difference in the zero-point correction from only the optical phonons, or by the negligible contribution of the rest of the modes (Δ*F* < ±1 meV), it is convincing to assign the main effect in the relative stabilization/destabilization of tetrahedral vs. octahedral site to the high-frequency end of the spectrum Fig. 5 shows the evolution of this difference with T when all modes are included in Equation (4). We notice that for high temperatures the tetrahedral site is further stabilized compared to the octahedral one.

## Discussion

We benchmark our model for the free energy by computing the diffusion coefficient for an interstitial H jumping from a tetrahedral site in $\{\frac{1}{8},\frac{1}{4},0\}$ to another tetrahedral site $\{\frac{1}{4},\frac{1}{8},0\}$, via a transition state located near {0.19, 0.19, 0}, with a computed barrier of Δ*U*(*TS* − *t*) = 109 meV (total internal electronic energy only). An expression valid for $T < w\lesssim 3000$ K, where *w* represents the highest optical phonon, is^33^:

1 $$D(T)=f\frac{4}{6}{a}^{2}\frac{{k}_{B}T}{h}{e}^{-\frac{{\rm{\Delta }}F(T)}{{k}_{B}T}}(\frac{{{\rm{m}}}^{2}}{{\rm{s}}})$$

where Δ*F*(*T*) > 0 is the total free energy barrier between the transition state and the absolute minimum (tetragonal site), *a* is the length of the jump, and *f* is a phenomenological factor representing the reduced mobility due to traps and other defects.

To evaluate Equation (1), and to produce an usable expression for the diffusion of interstitial hydrogen in *α*-iron, we take for *a* a typical value for the distance between tetragonal sites, *a* ≈ 1 Å. Regarding *f*, it depends in general on the number of traps and their trapping energy^19,23,34^; here we use a constant value, $f=\frac{1}{2}$, which yields an overall best agreement in the range of temperatures of interest between experiments and DFT ab-initio computed values. Δ*F*(*T*) is obtained using Equation (4) for the tetragonal and octahedral sites. As commented above, the calculation of converged phonons at the transition state is quite involved; therefore, we approximate the phonons at the transition state by those corresponding to the neighbouring symmetrical octahedral site. Since phonons involve smaller energies than typical energies in chemical bonds, their calculation using DFT implies more stringent conditions on the convergence thresholds. In that regard, conditions derived from imposing a symmetry group facilitate the process of finding self-consistency and convergence of energies and forces, because symmetry helps to compensate small round errors for slightly different but equivalent geometrical configurations. The resulting variation with temperature of Helmholtz’s free energy, Equation (4), is shown in Fig. 5. As the temperature increases, the effective barrier increases by about 15% because of the differences in the phonon spectra between the tetrahedral and octahedral sites. Therefore, we obtain the following parametrization to our full ab-initio calculations, valid over the temperature range $300\lesssim T\lesssim 850$ K (at Curie’s temperature, *T*_*C*_ = 1043 K, iron adopts a paramagnetic phase different from the ferromagnetic one assumed in these calculations; furthermore, at T^*^ = 1185 K, iron undergoes a phase transition to a face-centered cubic structure: as these temperatures are approached, the assumptions underlying the model become less valid).

2 $$D(T)=0.156T{e}^{-\frac{453}{T}(1+{({10}^{-3}T)}^{2}+{({10}^{-3}T)}^{4})}\,{10}^{-9}(\frac{{{\rm{m}}}^{2}}{{\rm{s}}})$$

where *T* is in Kelvin.

**Figure 6:**
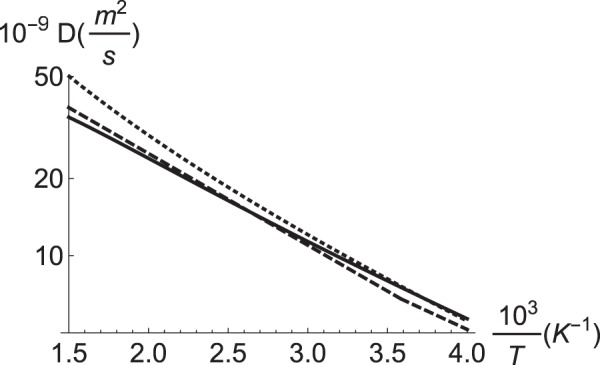
Arrhenius plot for the diffusion coefficient 10^−9^ *D* (m^2^/s) vs the inverse temperature $\frac{1000}{T}$ (K^−1^). Continuous: result based in Equation (1). Dashed: experimental data taken from^20^. Dotted: DFT theoretical result taken from^18^. Notice that the correction $f=\frac{1}{2}$ has been applied equally to both ref.^18^ and this work.

The behaviour of this expression is displayed as the continous line in the Arrhenius plot in Fig. 6. The dashed line represents an extensive compilation of experimental results, as selected by Kiuchi and MacLellan^20^; these authors have carefully analysed an extensive experimental dataset to report a representative value for the diffusion barrier of ≈60 meV for *T* < 300 K and ≈70 meV for *T* > 300 K. In addition, for the sake of comparison, we include a similar theoretical result by Jiang and Carter, where the total electronic energy has been corrected by the zero-point energy, yielding a barrier independent of temperature of ≈40 meV (dotted line)^18^. Comparing with such a DFT approach, the effect of phonons introduces a correction on the diffusion coefficient of about 67% towards the high temperature region, improving the agreement with the experimental dataset because the phonon-corrected barrier goes approximately from 45 meV at *T* = 300 K to 60 meV at *T* = 600 K. To quantify the improvement in the theory-experiment agreement, we compute a simple figure of merit, $R={\int }_{{T}_{1}}^{{T}_{2}}\,{10}^{9}|{D}_{thr}(T)-{D}_{exp}(T)|dT$, which behaves like a metric and goes to zero when the pair of functions converge uniformly to each other. In the interval of temperatures of interest, i.e. between 300 and 850 K, we find for the conventional DFT approach (dotted line) *R* = 12.2, and for the present approach that includes the phonons in the free energy (continuous line) *R* = 3.1. Therefore, these R values confirm the better tendency of theory towards high temperatures. We remark that we restrict ourselves to the region 300 < *T* < 850 K; for higher temperatures iron starts to approch a different phase, while for lower temperatures other effects should be taken into account, like tunnelling-aided diffusion. In our calculations, we do not interpret the change in the diffusion barrier between high and low temperature as a transition between two different regimes, as it is assumed in ref. ^20^ Instead, we associate the difference in the diffusion-barrier with a continuous variation due to the population of vibrational modes with increasing T. We notice that this is a result that we expect to be independent of the approximation we have made to substitute the phonons at the transition state by the ones at the local maximum at the octahedral site. From this result, we conclude that the contribution of phonons is almost entirely responsible for the former discrepancy between experiment and theory observed in the region of high-temperatures. The discrepancy between theory and experiment for low temperatures is likely related to H tunnelling assisted diffusion. For such a mechanism we refer to the path integral calculation by Kimizuka *et al*., that is better suited to describe the region *T* < 300 K^35^.

## Conclusion

In conclusion, these results help to identify a physical mechanism that is relevant to describe the diffusion of interstitials; the contribution of phonons to the free energy. These calculations prove that the contribution to Helmholtz’s free energy of phonons is enough to improve the description of interstitial diffusion in a range of temperatures between 300 and 850 K, which are relevant for industrial and civil engineering applications. Furthermore, these results suggest an experimental route to confirm the specified role of phonons via the computed *C*_*V*_(*T*), or the predicted softening of the speed of sound.

## Methods

### Density functional theory

**Figure 7:**
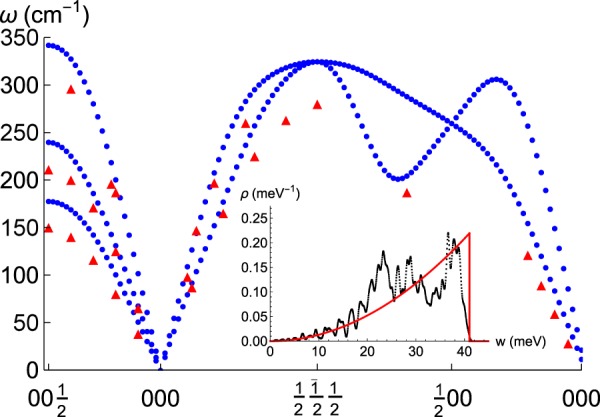
Benchmark calculation: Phonon dispersion in the first Brillouin zone for *α*-Fe along the path $00\frac{1}{2}$ (N) - 000 (Γ) - $\frac{1}{2}\frac{1}{2}\frac{1}{2}$ (H) - $\frac{1}{2}00$ (P) - 000 (Γ). Red triangles are experimental data^45^. Inset: Corresponding density of states. Integration smearing width is 0.25 meV. MP grid is such that Δ*k*_*i*_ < 0.025 Å^−1^. Black: DFT. Red: Debye approximation with *T*_*D*_ = 476 K. ${\int }_{0}^{\infty }\,\rho (w)dw=3$.

We utilize spin-polarized ab-initio Density Functional Theory, as implemented in CASTEP (plane-waves basis)^36^. Fe and H have been described by norm-conserving on-the-fly pseudopotentials (C. J. Pickard; H: 1*s* 1|0.8|11|15|18|10*N*(*qc* = 8)[]; Fe: 3*s*^2^ 3*p*^6^ 3*d*^6^ 4*s*2 3|1.3|35|45|48|30*N*:40*N*:31*N*:32*NN*(*qc* = 10.5)[]) with Koelling-Hamon relativistic corrections^37^. At a microscopic level, magnetism derives from spin, a quantum operator that can only be properly justified in a relativistic theory, i.e. using Dirac’s Equation. Already for H, a simple estimate based in Heisenberg’s Uncertainty Principle results in an *effective* velocity for the electron of about one-tenth of the speed of light. As the nucleus grows, the electrostatic interaction with the inner electron shells grows accordingly and makes relativistic corrections more important. This physical effect is well known, although usually it is ignored in favour of faster standard non-relativistic methods. However, Koelling and Hamon devised a technique easy to implement in standard band-structure calculations, and at the same time able to include part of the desired relativistic corrections. A 1300 eV energy cutoff and a 5 × 5 × 5 Monkhorst-Pack (MP) grid has been used to sample wavefunctions in the Brillouin zone^38^. The exchange and correlation energy has been computed within the Perdew-Burke-Ernzerhof generalized-gradient approximation (PBE)^39^. Thresholds for converged geometry configurations are: 10^−8^ eV for internal electronic total energies, 10^−3^ eV/Å for the maximum residual force, 0.01 GPa for the residual stress and 10^−4^ Å for the maximum displacement of atoms in the last three iterations (the window for convergence). By using these parameters, we find that total energy for optimal geometries can be given with a precision of ≈±5 meV. For the calculation of phonons, the internal electronic total energy has been converged further to 10^−10^ eV. Geometry optimisations have been performed using the BFGS minimiser^40^. Phonons have been computed from a finite displacement method based on a supercell defined by a cutoff radius of 4.5 Å, and a smearing width of 0.25 meV has been used to obtain the phonon density of states. The transition state has been computed using a quadratic synchronous transit method^41^. Such a formalism yields a reasonable approach to the phonons of pristine *α*-Fe^42,43^, as it is well documented in the literature, and as it is visualized e.g. in Fig. 7. The phonon density of states in this figure has been used to compute thermodynamical magnitudes for clean Fe.

### Thermophysics

Vibrations of atoms in the solid have been modelled as a set of independent harmonic oscillators with a characteristic dispersion relation, ${w}_{\overrightarrow{k}}$, where $\overrightarrow{k}$ takes values in the first Brillouin zone. These phonons have been obtained from the ab-initio DFT calculation, as explained above. In the canonical ensemble (NVT), the partition function of this system is^44^:

3 $${Z}_{w}(N,V,T)={{\rm{\Pi }}}_{k}\frac{{e}^{-\frac{\hslash {w}_{k}}{2{k}_{B}T}}}{1-{e}^{-\frac{\hslash {w}_{k}}{{k}_{B}T}}}={{\rm{\Pi }}}_{k}\,{z}_{w}(T,{w}_{k}),$$

where the number of particles (*N*) is fixed, and the volume (*V*) corresponds to a zero external stress condition.

All the thermodynamic magnitudes of interest can be obtained from the partition function. In particular, our primary interest is to get Helmholtz’s free energy:

4 $$\begin{array}{ccc}{F}_{w}=-\,{k}_{B}T\,\mathrm{ln}({Z}_{w}) & = & -{k}_{B}T\sum _{k}\,\mathrm{ln}\,{z}_{w}(T,{w}_{k})={k}_{B}T\sum _{k}\,\mathrm{ln}(2\,\sinh \,\frac{\hslash {w}_{k}}{2{k}_{B}T})\\  & = & {k}_{B}T{\int }_{0}^{\infty }\,\mathrm{ln}(2\,\sinh \,\frac{\hslash {w}_{k}}{2{k}_{B}T})\rho ({w}_{k})d{w}_{k},\end{array}$$

the sum is substituted by an integral in the first Brillouin zone using the phonon density of states, *ρ*(*w*_*k*_), that obeys the sum rule:

5 $${\int }_{0}^{\infty }\,\rho ({w}_{k})dw=3N$$

Since *F*_*w*_ is the characteristic function for the independent variables *N*, *V*, *T* we have:

6 $$d{F}_{w}=-\,{S}_{w}dT-pdV+\sum _{\alpha }\,{\mu }_{\alpha }d{N}_{\alpha },$$

which allows us to obtain other thermodynamic magnitudes of interest. The entropy, *TS*_*w*_:

7 $$-T{S}_{w}=-\,T{(\frac{\partial {F}_{w}}{\partial T})}_{V,N}={k}_{B}T\sum _{i}\,(\frac{\frac{\hslash {w}_{i}}{{k}_{B}T}}{1-{e}^{-\frac{\hslash {w}_{i}}{{k}_{B}T}}}+\,\mathrm{ln}(1-{e}^{-\frac{\hslash {w}_{i}}{{k}_{B}T}})),$$

the internal energy,

8 $${U}_{w}={F}_{w}+T{S}_{w}={k}_{B}{T}^{2}{(\frac{\partial {Z}_{w}}{\partial T})}_{V,N}=\sum _{i}\,\hslash {w}_{i}(\frac{1}{2}+\frac{1}{{e}^{\frac{\hslash {w}_{i}}{{k}_{B}T}}-1}),$$

or the specific heat at constant volume:

9 $${C}_{V}={(\frac{\partial {U}_{w}}{\partial T})}_{V,N}={k}_{B}\sum _{i}\,{(\frac{\hslash {w}_{i}}{{k}_{B}T})}^{2}\frac{{e}^{\frac{\hslash {w}_{i}}{{k}_{B}T}}}{{({e}^{\frac{\hslash {w}_{i}}{{k}_{B}T}}-1)}^{2}}$$

## References

[1] Elices M (2012). Failure analysis of prestressed anchor bars. Eng. Fail. Analysis.

[2] Elices M, Caballero L, Valiente A, Ruiz J, Martin A (2008). Hydrogen embrittlement of steels for prestressing concrete: The fip and dibt tests. Corros..

[3] Barrera O (2018). Understanding and mitigating hydrogen embrittlement of steels: a review of experimental, modelling and design progress from atomistic to continuum. J. Mater. Sci..

[4] Traidia A, Chatzidouros E, Jouiad M (2018). Review of hydrogen-assisted cracking models for application to service lifetime prediction and challenges in the oil and gas industry. Corros. Rev..

[5] Dwivedi SK, Vishwakarma M (2018). Hydrogen embrittlement in different materials: A review. Int. J. Hydrog. Energy.

[6] Larrosa NO, Akid R, Ainsworth RA (2018). Corrosion-fatigue: a review of damage tolerance models. Int. Mat. Rev..

[7] Diaz A, Alegre J, Cuesta I (2016). A review on diffusion modelling in hydrogen related failures of metals. Eng. Fail. Analysis.

[8] Oriani RA (1972). Mechanistic theory of hydrogen embrittlement of steels. Berichte Der Bunsen-Gesellschaft Fur Physikalische Chemie.

[9] Johnson WH (1875). On some remarkable changes produced in iron and steels by the action of hydrogen acids. Proc. R. Soc. Lond..

[10] Robertson IM (2015). Hydrogen embrittlement understood. Metall. Mater. Transactions B.

[11] Xu Q, Zhang J (2017). Novel methods for prevention of hydrogen embrittlement in iron. Sci. Reports.

[12] Iordachescu M, Valiente A, Perez-Guerrero M, Elices M (2018). Environment-assisted failure of structural tendons for construction and building applications. Constr. Build. Mater..

[13] Martínez-Pañeda E, Deshpande VS, Niordson CF, Fleck NA (2019). The role of plastic strain gradients in the crack growth resistance of metals. J. Mech. Phys. Solids.

[14] Castedo A, Sanchez J, Fullea J, Andrade MC, de Andres PL (2011). Ab initio study of the cubic-to-hexagonal phase transition promoted by interstitial hydrogen in iron. Phys. Rev. B.

[15] Sanchez J, Fullea J, Andrade MC, de Andres PL (2010). Ab initio molecular dynamics simulation of hydrogen diffusion in a-iron. Phys. Rev. B.

[16] Sanchez J, Fullea J, Andrade C, de Andres PL (2008). Hydrogen in *α*-iron: Stress and diffusion. Phys. Rev. B.

[17] Ramasubramaniam A, Itakura M, Carter EA (2009). Interatomic potentials for hydrogen in *α*–iron based on density functional theory. Phys. Rev. B.

[18] Jiang DE, Carter EA (2004). Diffusion of interstitial hydrogen into and through bcc fe from first principles. Phys. Rev. B.

[19] Turnbull A (2015). Perspectives on hydrogen uptake, diffusion and trapping. Int. J. Hydrog. Energy.

[20] Kiuchi K, McLellan RB (1983). The solubility and diffusitivy of hydrogen in well-annealed and deformed iron. Acta Met..

[21] Raina A, Deshpande V, Fleck N (2018). Analysis of thermal desorption of hydrogen in metallic alloys. Acta Materialia.

[22] Schaller R, Scully J (2014). Measurement of effective hydrogen diffusivity using the scanning kelvin probe. Electrochem. Commun..

[23] Li D, Gangloff R, Scully J (2004). Hydrogen trap states in ultrahigh-strength aermet 100 steel. Metall. Mater. Transactions a-Physical Metall. Mater. Sci..

[24] Hohenberg P, Kohn W (1964). Inhomogeneous electron gas. Phys. Rev..

[25] Kohn W, Sham LJ (1965). Self-consistent equations including exchange and correlation effects. Phys. Rev..

[26] Vanderbilt D (1990). Soft self-consistent pseudopotentials in a generalized eigenvalue formalism. Phys. Rev. B.

[27] Hamann DR, Schlüter M, Chiang C (1979). Norm-conserving pseudopotentials. Phys. Rev. Lett..

[28] Martin, R. M. *Electronic Structure: Basic Theory and Practical Methods*. Chap. 11 (Cambridge University Press, 2004).

[29] Leonov I (2014). Electronic correlations determine the phase stability of iron up to the melting temperature. Sci. Rep..

[30] Lide, D. R. (ed.) *Handbook of Chemistry and Physics*, 88th edn (CRC, London, 2007).

[31] Frisch, M. J. *et al*. Gaussian-09 Rev. E.01. Model: B3LYP; H(cc-pVTZ), Fe(sdd).

[32] Adams JJ, Agosta DS, Leisurea RG (2006). Elastic constants of monocrystal iron from 3 k to 500 k. J. Appl. Phys..

[33] Kehr K. W. (1978). Theory of the diffusion of hydrogen in metals. Topics in Applied Physics.

[34] Oriani RA (1970). Diffusion and trapping of hydrogen in steel. Acta Metall..

[35] Kimizuka H, Mori H, Ogata S (2011). Effect of temperature on fast hydrogen diffusion in iron: A path-integral quantum dynamics approach. Phys. Rev. B.

[36] Segall MD (2005). First-principles methods using castep. Z. fuer Kristallographie.

[37] Koelling DD, Harmon BN (1977). A technique for relativistic spin-polarised calculations. J. Phys. C: Solid State Phys..

[38] Monkhorst HJ, Pack JD (1976). Special points for brillouin-zone integrations. Phys. Rev. B.

[39] Perdew JP, Burke K, Ernzerhof M (1996). Generalized gradient approximation made simple. Phys. Rev. Lett..

[40] Pfrommer BG, Cote M, Louie SG, Cohen ML (1997). Relaxation of crystals with the quasi-newton method. J. Comput. Phys..

[41] Govind Niranjan, Petersen Max, Fitzgerald George, King-Smith Dominic, Andzelm Jan (2003). A generalized synchronous transit method for transition state location. Computational Materials Science.

[42] Minkiewicz VJ, Shirane G, Nathans R (1967). Phonon dispersion relation for iron. Phys. Rev..

[43] Sasaki T, Rappe AM, Louie SG (1995). Ab initio optimized pseudopotential calculations of magnetic systems. Phys. Rev. B.

[44] Dove, M. T. *Introduction to Lattice Dynamics*. (Cambridge U. Press, Cambridge (UK), 1993).

[45] Klotz S, Braden M (2000). Phonon dispersion of bcc iron to 10 Gpa. Phys. Rev. Lett..

